# Linking chemical-composition to antimicrobial efficacy: development of an essential oil-based topical gel prototype

**DOI:** 10.1007/s00253-025-13650-8

**Published:** 2025-12-01

**Authors:** Narjis Aftab, Pooja Varghese, Ammara Khalid, Aisha Kiro Umar, Christopher J. Wallis, Matt Bates, Sarah E. Hooper

**Affiliations:** 1 Microbiology and Infection Research Group, Cardiff School of Sport and Health Sciences, Cardiff Metropolitan University, Cardiff, CF5 2YB UK; 2 ZERO2FIVE Food Industry Centre, Cardiff Metropolitan University, Cardiff, CF5 2YB UK; 3 MCBA Consulting Ltd., Cardiff, UK; 4 Centre for Health, Immunology, Microbiology, and Environment (CHIME), Cardiff Metropolitan University, Cardiff, CF5 2YB UK

**Keywords:** Bactericidal, *Lamiaceae*, Antimicrobial, TD-GCMS

## Abstract

**Abstract:**

Antimicrobial resistance (AMR) is problematic for the management of chronic wound infection, where biofilms confer increased tolerance to treatments. A wealth of research describes the antimicrobial activity of essential oils, but none have been formulated for clinical use. We screened ten commercially available essential oils from the *Lamiaceae* plant family (thyme, rosemary, basil, oregano, clary sage) for bacteriostatic, bactericidal, and anti-biofilm activity. TD-GCMS was used to identify highly abundant compounds which were mapped to efficacy data. Thyme essential oils were antimicrobial against both *Pseudomonas aeruginosa* and *Staphylococcus aureus* and had the most potent anti-biofilm activity. Three compounds were common and highly abundant in these oils: *o*-cymene, 2-isopropyl-4-methyl-phenol (*o*-thymol/carvacrol), and thymol. The most efficacious red and white thyme oils were formulated into Laponite-based hydrogel emulsions capable of inhibiting both *P. aeruginosa* and *S. aureus* in static and dynamic biofilm models*.* Notably, the efficacy of both gels diverged from that predicted by MIC, MBC, and MBIC values, highlighting the limitations of reductionist analyses in predicting real-world antimicrobial performance.

**Key points:**

• *Thyme oils are the most efficacious of the Lamiaceae plant oils tested*

• *Thymol isomers and o-cymene are abundant in thyme oils, but minor components also play a role in antimicrobial activity*

• *Hydrogel efficacy arises from interactions between formulation and wound microenvironment*

## Introduction

Wound infections affect more than 3.8 million people in the UK, a rate comparable to diabetes and cardiovascular disease (Guest et al. 2020). Despite this, wound infection is overlooked as a medical crisis. One of the biggest challenges in modern-day treatment of chronic wound infection is antimicrobial resistance (Blackburn et al. 2025). With few new antimicrobials in development, nature’s innovations offer potential; still only manuka honey has reached clinical use, becoming the first medical-grade natural product for wound treatment in 1999 (Cooper and Gray, 2012).

Plant-based remedies have been used for centuries in traditional medicine systems with anecdotal evidence describing antimicrobial activity (Spellberg et al. 2025). Numerous research studies clarify these observations, and chemical analyses reveal complex mixtures of “bioactive” compounds, such as polyphenols and terpenoids, which contribute to these therapeutic effects (Abdullah et al. 2024; Mssillou et al. 2025; Pacyga et al. 2024; Vaou et al. 2021). Essential oils extracted from plants in the *Lamiaceae* family, which includes basil, oregano, rosemary, clary sage, and thyme, to name a few, have consistently demonstrated potent antimicrobial and anti-inflammatory activities (Alsakhawy et al. 2022; Avasiloaiei et al. 2023; Marques et al. 2023; Mssillou et al. 2025).

Although in vitro results are encouraging, essential oils are not used clinically to treat infection, in part due to insufficient human data, variable outcomes in animal models, potential safety concerns, and a lack of standardised extraction and testing protocols (Hulankova 2022). The volatility and poor bioavailability of essential oils also present challenges for their delivery (Folle et al. 2024). To overcome these translational hurdles consideration must be given to optimal chemical composition and delivery.

To address these challenges, we screened ten essential oils from the *Lamiaceae* family for antimicrobial, anti-biofilm, and bactericidal activity against two wound pathogens, *Pseudomonas aeruginosa* and *Staphylococcus aureus.* The aim was to determine highly abundant compounds associated with antimicrobial and antibiofilm activity and, establish whether the most potent oils could be formulated into a topical hydrogel.

## Materials and methods

### Bacterial strains and culture conditions

*Pseudomonas aeruginosa* American Type Culture Collection (ATCC) 9027 was used because it is a widely accepted and commercially available reference stain used for sterility and antimicrobial efficacy testing; it is used routinely for the study of wound infection and antimicrobial testing in this context (Jayal et al., 2010); Raizman et al. 2021). *Staphylococcus aureus* National Collection of Type Cultures (NCTC) 13616 (Epidemic Methicillin Resistant *S. aureus* -15; EMRSA-15) was used because it is a reference strain (*mecA* positive and *mecC* negative) with high prevalence in acute and chronic wounds (Hart et al. 2014; Udo et al. 2006). Both bacteria were routinely cultured in nutrient broth (NB) and maintained on nutrient agar (NA; Sigma-Aldrich, UK) at 37 °C. Cultures were in the exponential phase and equilibrated prior to each experiment.

### Preparation of oils

Oils were purchased from commercial suppliers and stock concentrations were prepared at 600 mg/mL in 40% (v/v) ethanol (Sigma-Aldrich, Gillingham, UK), stored at room temperature, and protected from light (Table 1). Red thyme oils were prepared at 600 mg/mL in 10% (v/v) dimethyl sulfoxide (DMSO; Sigma-Aldrich, UK) and stored as described. Supplier names are not disclosed as some products were found to differ from their stated identity as 100% pure essential oil; however, all essential oils were chemically characterised, ensuring reproducibility based on verified composition rather than brand source.

**Table 1:** Essential oils used in this study. Plant species, parts and extraction details are given. For clary sage, rosemary and red thyme, (A) and (B) denote different suppliers of the same named oil, marketed as 100% essential oil. For white thyme: (A) and (B) denote two different species of the genus *Thymus*

Oil	Species	Plant parts	Extraction
Basil	*Ocimum basilicum*	Flowers	Steam distillation
Clary sage (A)	*Salvia sclarea*	Whole plant	Steam distillation
Clary sage (B)	*Salvia sclarea*	Whole plant	Steam distillation
Oregano	*Origanum vulgare*	Leaves and flowers	Steam distillation
Rosemary (A)	*Salvia rosmarinus*	Leaves and flowers	Steam distillation
Rosemary (B)	*Salvia rosmarinus*	Leaves and flowering tips	Steam distillation
Red thyme (A)	*Thymus vulgaris*	Leaves	First distillation
Red thyme (B)	*Thymus vulgaris*	Leaves and seeds	First distillation
White thyme (A)	*Thymus vulgaris*	Leaves	Second distillation
White thyme (B)	*Thymus zygis*	Entire plant	Steam distillation

### Minimum inhibitory concentration (MIC)

These experiments followed the protocol of Devienne and Raddi (Devienne and Raddi 2002) for screening natural products using a microplate photometer. Pre-cultures of bacteria were equilibrated to an OD of 0.05 (A_650_) (equivalent to 4 × 10^7^ cfu/mL) and inoculated (10 µL) into prepared serial doubling dilutions (0–300 mg/mL) of each oil in a sterile 96-well microtitre plate (Greiner Bio-one, UK) in NB, in a total volume of 100 µL. Vehicle controls established that neither solvent (ethanol at 0–20% v/v range or DMSO at 0–5% v/v range) inhibited bacterial growth nor resulted in bacterial death, which concurs with published studies (Chambers et al. 2006; Elzain et al. 2019; Wanigasekara et al. 2021) . After 16 h incubation at 37 °C the MIC was determined by spectrophotometry using a SpectoStar Nano (BMG Labtech Ltd., UK) microtitre plate reader at A_650_ and defined as the lowest concentration showing no visible growth. Negative controls comprised NB broth only; positive controls containing no oil were inoculated with bacteria as described.

### Minimum bactericidal concentration (MBC)

These experiments adhered to Clinical Laboratory Standards Institute guidelines (CLSI M07; https://clsi.org/shop/standards/m07/) but substituted MHB/MHA with NB/NA for consistency with the MIC protocol described above. Based on the MIC results, 5 µL culture (taken from wells corresponding to MIC, ½ × MIC, 2 × MIC) was spotted onto NA and incubated at 37 °C for 16 h. MBC was defined as the lowest concentration showing no visible growth.

### Minimum biofilm inhibition concentration (MBIC)

Biofilms were cultured in 96-well microtitre plates (Greiner Bio-one, UK)., prepared and incubated as described for MIC. Media and planktonic cells were aspirated and discarded. Biofilms were washed 5 times with sterile phosphate-buffered saline (PBS; Sigma-Aldrich, UK) and stained with crystal violet (0.25% w/v in water; Sigma-Aldrich, UK) for 5 min at room temperature. The crystal violet was aspirated and discarded, and biofilms were washed five times with sterile PBS. To re-solubilise the bound crystal violet, 100 µL of acetic acid (7% v/v; Sigma-Aldrich, UK) was added to each well and the colour intensity was analysed using a SpectroStar Nano microtitre (BMG Labtech Ltd., UK) plate reader at A_595_. The MBIC was defined as the lowest concentration that inhibited biomass accumulation; biofilm reduction was determined as a percent difference in biomass compared to the untreated control (Macia et al. 2014).

### TD-GCMS analysis of essential oil samples

For analysis, 5 µL of each essential oil was diluted into 20 mL of HPLC-grade methanol (Fisher Scientific, UK) in 20 mL headspace vials to minimise any headspace. Vials were sealed with screw-on caps fitted with PTFE-backed septa. A 4 µL aliquot of each diluted solution was then injected, in a 50 mL flow of high-purity nitrogen (N₂), onto a short-bed (1 cm) Tenax TA, glass thermal desorption tube (CAMSCO Inc., USA) using a CSLR liquid injection system (Markes International, UK). Following loading, the tubes were purged with nitrogen for 2 min (100 mL total volume) to allow the methanol solvent to break through while retaining the target volatile and semi-volatile compounds present in each essential oil. Tubes were then sealed with brass caps fitted with PTFE ferrules and stored at ambient temperature until analysis.

Sample analysis was performed using a TD100 thermal desorption unit (Markes International, UK) connected to an Agilent 6890 gas chromatograph and an Agilent 5973 mass spectrometer (Agilent Inc., USA). Tubes were desorbed at 280 °C for 7 min with a helium flow of 50 mL/min, and analytes were re-focused on a custom-packed cold trap containing quartz wool, Tenax TA, and SulfiCam (CAMSCO Inc., USA), maintained at 25 °C. The trap was then rapidly heated to 280 °C (maximum heating rate) with a split flow of 25 mL/min and held for 3 min, transferring analytes in a narrow band onto the GC column. The TD100 flow path, including the heated valve and transfer line, was maintained at 200 °C throughout the analysis.

A HP-5MS capillary column (30 m × 0.25 mm i.d. × 0.25 µm film thickness; Agilent Inc., USA) was used for chromatographic separation. The GC was operated in constant flow mode (2 mL/min helium). The oven temperature program was as follows: initial temperature 30 °C (2 min hold), ramped at 20 °C/min to a final temperature of 280 °C (5 min hold).

The Agilent 5973 mass spectrometer was operated in electron ionization (EI) mode at 70 eV. Data were acquired in full scan mode over the range m/z 35–400. The ion source temperature was maintained at 230 °C, the quadrupole at 150 °C, and the transfer line at 280 °C. ChemStation data files were converted to MassHunter format using the GC MSD Translator software (Agilent Inc., USA). MassHunter Unknowns Analysis (Agilent Inc., USA) was then used to tentatively identify compounds by comparing mass spectra with those in the NIST/EPA/NIH Mass Spectral Library (version 2023), applying a minimum match factor threshold of ≥ 75%.

The elution order of the tentatively identified compounds, including many terpenes, was examined and adjusted in each sample to ensure consistency across the dataset. Where available, compound identities were further verified against authentic reference standards. The analyses are semi-quantitative, in that each essential oil was diluted and injected in an identical way, meaning that all results are directly inter-comparable. Quality control measures included the analysis of solvent blanks, system blanks, and replicate injections. Retention time reproducibility was maintained within ± 0.05 min, and daily system suitability was verified using a standard reference mixture.

### Data analysis using ChromMine™

The combined output of all samples from MassHunter Unknowns Analysis (Agilent Inc., USA) was exported as a CSV file containing sample name, file name, compound name, peak area, CAS number, chemical formula, and match factor. This dataset was then imported into ChromMine™ (FuniSoft Ltd, UK), a cloud-based software platform for mining and interpreting complex chromatographic datasets.

To ensure data consistency, ChromMine™ checked for erroneous repeated compound identifications within single samples and across the dataset. Where compounds were identified more than once, the software grouped them within user-defined retention time (RT) windows. Each group was then automatically renamed with a median RT-based name to ensure that they were treated as distinct compounds during sample comparison. All corrections were applied automatically but remained subject to user review. Peak areas were normalised across the dataset, enabling semi-quantitative comparisons between essential oils.

Custom metadata fields were used to encode the observed biological activity of each essential oil (based on MIC and MBC). Clustering functions compared chemical profiles against biological efficacy and grouped oils accordingly.

### Hydrogel preparation

A hydrogel of 4.7% (w/v) Laponite RD (Blagden Speciality Chemicals Ltd., UK), dissolved in sterile distilled water was prepared by mixing with stirring on a magnetic plate set to a medium speed, for 10 min, at room temperature until it was completely dissolved. While there is no standard for laponite concentration in topical and cosmetic products, we have previously used 4.7% (w/v) as an effective delivery vehicle for wound antimicrobials (Khalid et al. 2023; Nedelea et al. 2021). The resulting gel was sterilised by autoclaving at 121 °C for 20 min. Two thyme oils (red thyme B and white thyme B, selected based on having the lowest MIC, MBC and MBIC), were added to separate laponite gel preparations, to achieve MIC, 2 × MIC, 4 × MIC, 6 × MIC, and 8 × MIC. Prepared stocks (600 mg/mL) of each oil were used and the volume added to achieve the required dose was such that it did not affect the concentration of the gel. Gels were mixed by stirring on a magnetic plate set to a medium speed, at room temperature for 10 min, to ensure even distribution of the oil. All gels had a final pH of 8.5 (laponite forms a gel at pH 8.3–9.8) and were stored at room temperature, protected from light.

### Zone of inhibition

Bacterial cultures were equilibrated to OD 0.1 (A_620_) (equivalent to 8 × 10^7^ cfu/mL) and 100 µL was used to prepare a spread plate onto NA. A sterile yellow pipette tip (7.5 mm outer diameter) was used to bore a hole in the centre of each lawn and fill it with 0.15 g gel. Laponite (4.7% w/v) in sterile distilled water was used as a vehicle control, and Neosporin topical wound cream (Kenvue Brands LLC, USA; bacitracin zinc 400U, neomycin sulphate 3.5 mg, polymyxin B sulphate 5000 U) was used as a positive control. Plates were incubated at 37 °C for 16 h, and zones of inhibition were measured in mm using digital callipers.

### Biofilm flow system

Four replicate biofilms were cultured in a custom flow system maintained at 33 °C (typical temperature of a chronic wound bed (Gethin et al. 2021)),with a flow rate of 0.322 mL/min (Duckworth et al. 2018). The full protocol is described in Duckworth et al (2018) and Khalid et al (2023) and available at Hooper Group: Biofilm Models. After 24 h culture, 0.15 g of gel was applied to the biofilm. At this point, two of the four replicate biofilms were enumerated by total viable count (TVC) to give a baseline measurement immediately following the application of treatment. After 48 h and 72 h, biofilms were harvested and enumerated by TVC as described in Khalid et al (2023).

## Results

### Inhibitory and bactericidal activity of the essential oils

Using a doubling dilution range of 0–300 mg/mL, MICs were confirmed for all oils against both bacteria (Table 2). MBCs were also determined using the same dilution range. No MBC was found for clary sage (A) for either bacterium. No MBC was found for rosemary (A) against *S. aureus*. Thyme oils had the lowest MICs and MBCs against both pathogens. Plant compounds are routinely classified as antimicrobials if their MICs range between 100 and 1000 µg/mL (Bubonja-Šonje et al. 2020). Therefore, only red thyme (A and B), white thyme (A and B) can be considered antimicrobial against *S. aureus* and only red thyme (B) can be considered antimicrobial against *P. aeruginosa*.

**Table 2:** Minimum inhibitory concentration (MIC) and minimal bactericidal concentration (MBC) of essentials oils against *S. aureus* and *P. aeruginosa*

Oil	Species	*P. aeruginosa*		*S. aureus*	
		MIC (mg/mL)	MBC (mg/mL)	MIC (mg/mL)	MBC (mg/mL)
Basil	*Ocimum basilicum*	150	150	75	300
Clary sage (A)	*Salvia sclarea*	75	No MBC	37.5	No MBC
Clary sage (B)	*Salvia sclarea*	75	150	18.8	37.5
Oregano	*Origanum vulgare*	37.5	75	4.7	4.7
Rosemary (A)	*Salvia rosmarinus*	150	150	75	No MBC
Rosemary (B)	*Salvia rosmarinus*	75	150	18.8	0
Red thyme (A)	*Thymus vulgaris*	1.2	2.4	0.6	0.6
Red thyme (B)	*Thymus vulgaris*	0.6	2.2	0.6	0.6
White thyme (A)	*Thymus vulgaris*	37.5	150	0.6	1.2
White thyme (B)	*Thymus zygis*	18.8	75	0.6	1.2

Despite this, using MBC/MIC ratios and a threshold of ≦4 to indicate bactericidal activity (Ishak et al. 2025), basil, clary sage (B), oregano, red thyme (A and B), and white thyme (A and B) were found to be bactericidal against both pathogens (Table 3).

**Table 3:** MBC/MIC ratios to establish whether the essential oils tested are bactericidal or bacteriostatic against *S. aureus* and *P. aeruginosa*. Values ≦4 indicate bactericidal activity

Oil	Species	*P. aeruginosa*	*S. aureus*
		MBC/MIC ratio	MBC/MIC ratio
Basil	*Ocimum basilicum*	1	4
Clary sage (A)	*Salvia sclarea*	N/A	N/A
Clary sage (B)	*Salvia sclarea*	2	2
Oregano	*Origanum vulgare*	2	1
Rosemary (A)	*Salvia rosmarinus*	1	N/A
Rosemary (B)	*Salvia rosmarinus*	2	8
Red thyme (A)	*Thymus vulgaris*	2	1
Red thyme (B)	*Thymus vulgaris*	4	1
White thyme (A)	*Thymus vulgaris*	4	2
White thyme (B)	*Thymus zygis*	4	2

### Antibiofilm activity of the essential oils

Red thyme (A and B) and white thyme (B) had the most potent antibiofilm activity against both bacteria (Table 4). There was no MBIC for basil, clary sage (A), oregano, or rosemary (A and B), for both bacteria. White thyme (A) did not produce an MBIC for *P. aeruginosa.* Where an MBIC was not achieved the biomass reduction ranged between 12.8 and 43.6% *S. aureus.*

**Table 4:** Minimum biofilm inhibition concentration (MBIC) of essentials oils against *S. aureus* and *P. aeruginosa*, including biomass reduction where MBIC was not achieved

Oil	Species	*P. aeruginosa*		*S. aureus*	
		MBIC (mg/mL)	Biomass (% reduction)	MBIC (mg/mL)	Biomass (% reduction)
Basil	*Ocimum basilicum*	No MBIC	28.5	No MBIC	12.8
Clary sage (A)	*Salvia sclarea*	No MBIC	19.6	No MBIC	21.5
Clary sage (B)	*Salvia sclarea*	150	100	18.8	100
Oregano	*Origanum vulgare*	No MBIC	29.8	No MBIC	34.2
Rosemary (A)	*Salvia rosmarinus*	No MBIC	15.7	No MBIC	19.9
Rosemary (B)	*Salvia rosmarinus*	No MBIC	43.6	No MBIC	28.5
Red thyme (A)	*Thymus vulgaris*	1.2	100	0.6	100
Red thyme (B)	*Thymus vulgaris*	0.6	100	0.6	100
White thyme (A)	*Thymus vulgaris*	No MBIC	32.5	0.6	100
White thyme (B)	*Thymus zygis*	1.2	100	1.2	100

### TD-GCMS of the essential oils

TD-GCMS was used to identify chemical components of each essential oil. A concentration of greater than 10% was taken to indicate compounds with the highest abundance, based on the recommendation of de Sousa et al. (De Sousa et al. 2023) (Table 5). The most abundant compounds in clary sage (A), oregano, rosemary (A) and white thyme (A) were isopropyl myristate and isopropyl palmitate which are emollients and texture enhancers used in the cosmetic industry. Clary sage (B), while containing isopropyl myristate, also contained linalool which is known to have antimicrobial activity (Tang et al. 2025). Rosemary (B) did not contain any emollients, but was found to have eucalyptol, 3-carene, and camphor in high concentrations, which are known antimicrobial compounds (Duda-Madej et al. 2024; Mączka et al. 2021; Tang et al. 2022). Oregano, red thyme oil (A) and white thyme oil (B) contained differing but high concentrations of thymol. All four thyme oils contained 2- isopropyl-4-methylphenol (*o*-thymol/carvacrol) between 16.4 and 65.76%, and red thyme oil (B) also contained 29.9% *o*-cymene. White thyme (A) was the least efficacious of the thyme oils and also contained both emollients at high concentrations.

**Table 5:** Highly abundant compounds identified by TD-GCMS and ChromMine™ analysis. Mean percentage composition is reported for each oil. [+ / −]  indicates standard deviation. Abundant compounds were taken to be those present at greater than 10%. Italicised compounds and values are emollients

Oil	Basil	Clary sage (A)	Clary sage (B)	Oregano	Rosemary (A)	Rosemary (B)	Red thyme (A)	Red thyme (B)	White thyme (A)	White thyme (B)
**Species**	***Ocimum basilicum***	***Salvia sclarea***	***Salvia sclarea***	***Origanum vulgare***	***Salvia rosmarinus***	***Salvia rosmarinus***	***Thymus vulgaris***	***Thymus vulgaris***	***Thymus vulgaris***	***Thymus zygis***
Anethole	23.9 [+ / − 0.1]									
Camphor						11.2 [+ / − 0.1]				
3-Carene						15.3 [+ / − 2.8]				
o-Cymene								29.9 [+ / − /1.9]		
Eucalyptol						34.7 [+ / − 0.3]				
2-Isopropyl-4-methylphenol							61.1 [+ / − 7.4]	46.8 [+ / − 0.4]	16.4 [+ / − 0.6]	65.7 [+ / − 3.8]
*Isopropyl myristate*	*55.6 [*+ / −*0.4]*	*44.3 [*+ / − *1.6]*	*20.1 [*+ / − *2.4]*	*38.7 [*+ / −*1.8]*	*42.6 [*+ / − *0.5]*				*46.5 [*+ / −*0.7]*	
*Isopropyl palmitate*		*31.3 [*+ / − *1.6]*		*27.1 [*+ / −*1.0]*	*32.4 [*+ / − *0.4]*				*33.9* *[*+ / −*1.9]*	
Linalyl acetate		40.3 [+ / − 1.9]								
Linalool			27.6 [+ / − 1.3]							
Thymol				27.2 [+ / − 0.2]			18.3 [+ / − 0.2]			14.7 [+ / − 0.7]

### Composition related to inhibitory activity

**Fig. 1:**
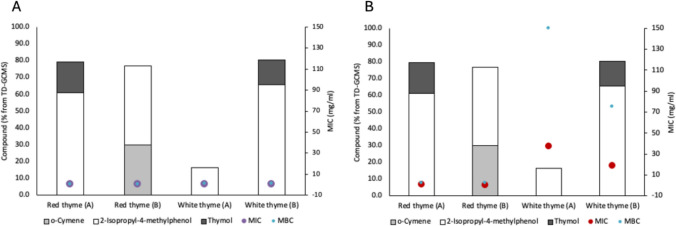
Thyme oil composition based on compounds present at > 10% composition, related to MIC and MBC. **A** For *S. aureus*; **B** for *P. aeruginosa*

Of the four thyme oils identified as antibacterial, the predominant compounds were mapped against MIC and MBC values (Fig. 1). In *S. aureus*, where all four thyme oils were regarded as antibacterial, 2-isopropyl-4-methylphenol (*o*-thymol/carvacrol) was a common compound (Fig. 1A). For *P. aeruginosa*, where only red thyme oil (Fig. 1B) was identified as antibacterial, *o*-cymene was uniquely abundant. The similar composition of red oil (A) and white oil (B), but their differing efficacy against *P. aeruginosa*, suggests that minor constituents also contribute to the observed antibacterial effect. It should be noted that TD-GC–MS identifies individual components but does not account for potential synergistic or antagonistic interactions that may influence overall efficacy.

**Fig. 2:**
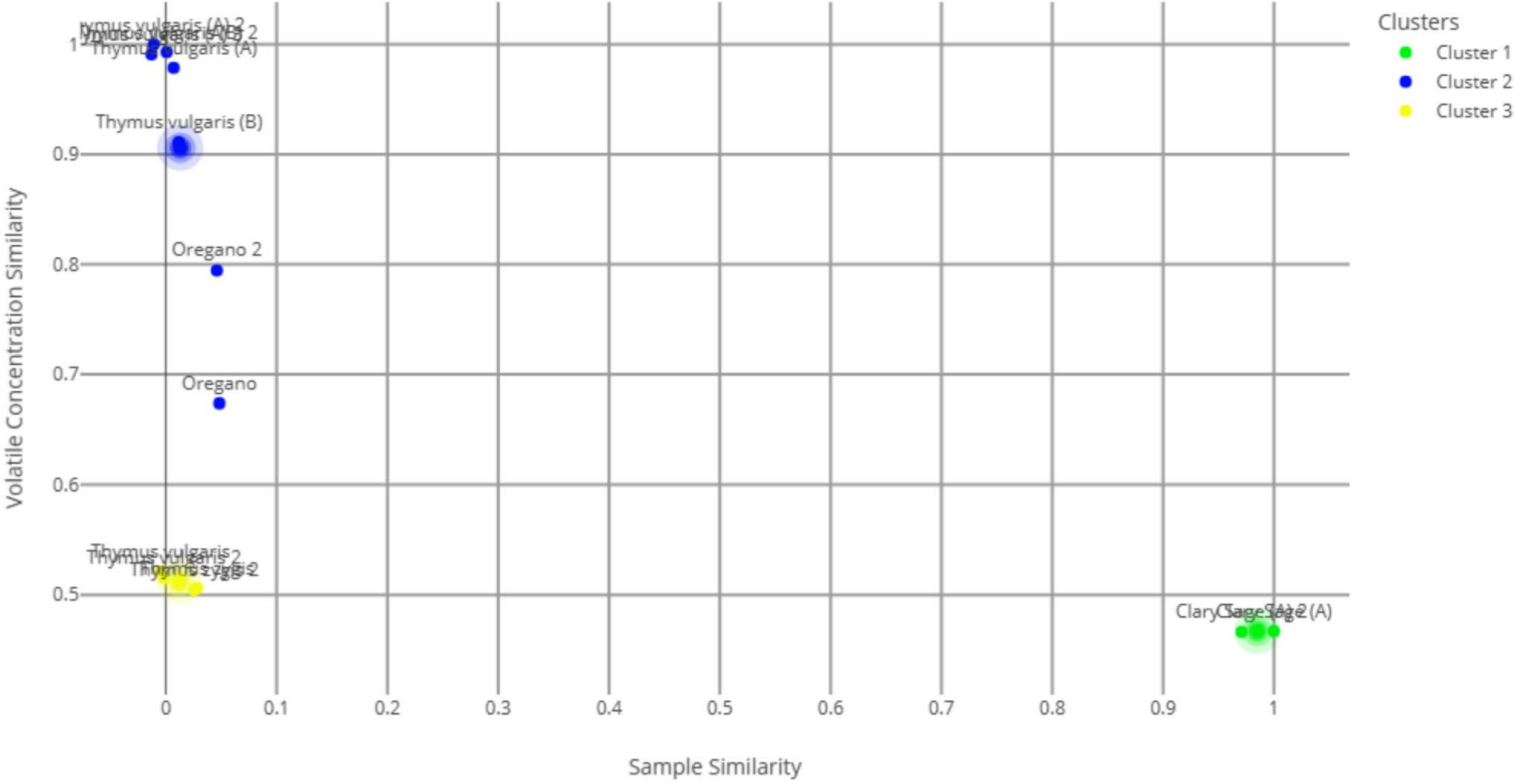
ChromMine.™ clustering analysis of essential oils based on integrated chemical composition and biological efficacy (MIC and MBC). Oils with the most potent bactericidal activity grouped separately: clusters 2 (thyme oils) and 3 (oregano oil) from those with weaker activity: cluster 1 (clary sage oil A)

Incorporating biological MIC/MBC data into ChromMine™, taking account of all major and minor chemical constituents, revealed distinct clustering of oils based on efficacy (Fig. 2). Cluster 1 (green) is a representative example of an oil (Clary sage (A)) that had no MBC for either bacterium and had a compound profile that contained no thymol or thymol isomers. Cluster 2 (blue) comprises red thyme oils (A and B), with oregano weakly associated with this cluster. These oils contain thymol or a thymol isomer, or both. Cluster 3 (yellow) represents white thyme oils (A and B). These oils clustered well based on activity and composition despite being from two different species; both oils contain either thymol or a thymol isomer. The different clustering despite similarities in the major compound profiles (abundance > 10%) might be the result of minor compounds (abundance between 0.1 and 9.9%) (De Sousa et al. 2023).

### Minor components of the essential oils assessed by TD-GCMS

TD-GCMS data for minor components (< 10% composition) revealed a varied chemical composition for all ten oils (Table 6); compounds present at below 0.1% were regarded as negligible (De Sousa et al 2023). No compounds were common to all oils, even within genera and species, and all chemical profiles were unique. Thymol isomers were present in clary sage (A), oregano, and rosemary (A), and *o*-cymene was present in red thyme (A) and white thyme (A). All minor compounds identified are reported to have antimicrobial activity (Angane et al. 2022).

**Table 6:** Minor components of all essential oils analysed by TD-GCMS. Mean percentage composition is reported; [ + / − ] indicated standard deviation. The minor compounds are known plant compounds, volatile organic acids, and reported to have antimicrobial activity

Oil:	Basil	Clary sage (A)	Clary sage (B)	Oregano	Rosemary (A)	Rosemary (B)	Red thyme (A)	Red thyme (B)	White thyme (A)	White thyme (B)
Species:	***Ocimum basilicum***	***Salvia sclarea***	***Salvia sclarea***	***Origanum vulgare***	***Salvia rosmarinus***	***Salvia rosmarinus***	***Thymus vulgaris***	***Thymus vulgaris***	***Thymus vulgaris***	***Thymus zygis***
Alphaguanidine		0.2								
Alpha-terpineol		3.6 [+ / − 0.1]			0.2	1.4 [+ / − 0.2]				
Anethole			0.4 [+ / − 0.1]	0.8 [+ / − 0.1]						
Beta-alaskene		0.8 [+ / − 0.1]								
Beta-myrcene				7.4 [+ / − 0.4]	0.2			0.6		
Beta-pinene		0.2	0.3							
Bornyl-acetate						1.5 [+ / − 0.4]				
Camalanene		1.0 [+ / − 0.1]								
Camphene		0.3 [+ / − 0.1]			0.4 [+ / − 0.1]			0.5 [+ / − 0.1]		
Camphor					6.2 [+ / − 0.3]					
Carene	0.5 [+ / − 0.1]									
3-carene		0.5 [+ / − 0.2]	1.5	0.2	5.2 [+ / − 0.2]		0.8 [+ / − 0.2]			
Carvone	0.3			0.2						
Caryophyllene			0.6		0.2		2.3 [+ / − 0.2]		0.9	
Cis-muurola-4(15),5-diene	0.4									
Cupranene		1.1 [+ / − 0.3]								
D-limonene	0.2		7.5 [+ / − 0.3]					0.6		
Endo-borneol					0.3	1.1 [+ / − 0.1]		0.5 [+ / − 0.2]		
Eucalyptol		5.6 [+ / − 0.2]					0.8 [+ / − 0.2]	0.5		1.4 [+ / − 0.1]
Eugenol	6.3			0.2 [+ / − 0.1]						
Gamma-terpinene		0.2				0.9 [+ / − 0.1]	0.9 [+ / − 0.1]			
Humulene						0.3 [+ / − 0.1]				
Linalool	6.9	3.0 [+ / − 0.3]			0.7		4.8 [+ / − 0.4]	4.5 [+ / − 0.1]		8.1 [+ / − 0.2]
Linalyl acetate		0.8 [+ / − 0.2]								
Nerol acetate			3.3 [+ / − 1.2]							
*o*-cymene							4.4 [+ / − 0.3]			3.2 [+ / − 0.8]
*p-*thymol				4.5 [+ / − 0.1]						
Seychelline		0.1								
Thymol	2.6	0.8 [+ / − 0.1]			0.2					
Trans-alphabergamotene			0.3 [+ / − 0.1]							
Verbenone					0.4 [+ / − 0.1		0.4			

### Efficacy of a laponite hydrogel emulsion of thyme oil

We formulated red thyme (B) and white thyme (B) into laponite gels based on their MIC, MBC, and MBIC for both bacteria. Our data suggested both gels should have efficacy against both bacteria, but that white thyme (B) gel should have better efficacy against *S. aureus.* In the zone of inhibition assays thyme (B) gel produced a zone of inhibition significantly greater (*p* < 0.05; one way ANOVA with Dunnett’s post hoc) than the Neosporin at 8 × MIC for *S. aureus* and 6 × and 8 × MIC for *P. aeruginosa* (Table 7)*.* White thyme (B) gel produced a zone of inhibition for both bacteria at all concentrations tested but was only significantly greater (*p* < 0.05; one way ANOVA with Dunnett’s post hoc) than the Neosporin control at 6 × MIC and greater (Table 7). This suggests that white thyme (B) gel is the most efficacious.

**Table 7:** Zone of inhibition for Laponite-thyme gels against *S. aureus* and *P. aeruginosa*. [EO] indicates the concentration of essential oil given as × MIC; [] denotes SEM. N (biological repeat) = 3, n (technical repeat) = 3

		**Zones of inhibition (mm)**		
**Organism**	**[EO]**	Red thyme (B)	White thyme (B)	Neosporin
		*T. vulgaris*	*T. zygis*	
***S. aureus***	MIC	0.0	8.16 [+/-0.01]	15.19
	2×MIC	0.0	8.59 [+/-0.18]	
	4×MIC	0.0	9.83* [+/-0.19]	
	6×MIC	9.11* [+/-0.24]	16.74* [+/-0.24]	
	8×MIC	15.14* [+/-0.16]	24.87* [+/-0.39]	
***P. aeruginosa***	MIC	0.0	9.66[+/-0.20]	7.09
	2×MIC	0.0	10.51[+/-0.50]	
	4×MIC	0.0	12.56* [+/-0.44]	
	6×MIC	11.22* [+/-0.43]	12.99a [+/-0.05]	
	8×MIC	12.32* [+/-0.85]	13.74b [+/-0.08]	

*Statistically significant difference (*p* < 0.05) compared to the Neosporin control

^a^Statistically significant difference (*p* < 0.05; one-way ANOVA with Tukey’s post hoc) from MIC and 2 × MIC, but not 4 × MIC

^b^Statistically significant difference (*p* < 0.05) from MIC, 2 × MIC and 4 × MIC, but not 6 × MIC (one-way ANOVA with Tukey’s post hoc)

### Testing thyme oil-laponite gels in an in vitro chronic wound biofilm model

Red and white thyme gels were formulated to contain 8 × MIC of essential oil and tested against both bacteria in a dynamic chronic wound biofilm model. Biofilms were established for 24 h prior to treatment. For *P. aeruginosa* at 48 h, (24 h post-treatment) the addition of red thyme (B) gel caused a statistically significant reduction (*p* < 0.05; one-way ANOVA with Dunnett’s post hoc) of 2.33 log. At 72 h, this was maintained at a 2.51 log reduction (Table 8). White thyme (B) gel caused a statistically significant reduction (*p* < 0.05; one-way ANOVA with Dunnett’s post hoc) of 2.94 log. At 72 h, this was maintained; the 0.54 log increase in TVC was not statistically significant (*p* > 0.05) when compared to the 48 h data (Table 8). For *S. aureus* treated with red thyme (B) gel, at 48 h (24 h post-treatment), there was a statistically significant reduction (*p* < 0.05; one-way ANOVA with Dunnett’s post hoc) of 2.36 log, which was maintained (2.20 log reduction) (Table 8). White thyme (B) gel resulted in a 2.71 log reduction at 48 h, maintained at 72 h, with an increase of 0.22 log that was not statistically significant (*p* > 0.05). Comparison of the efficacy of each gel for both bacteria revealed there was not a statistically significant (*p* < 0.05; one-way ANOVA with Tukey’s post hoc) difference between the reduction in bacterial load at each time point, indicating that despite differing chemical composition and MIC/MBC and MBIC values, both gels were effective in our model.

**Table 8:** Log_10_ CFU of *P. aeruginosa* or *S. aureus* recovered from biofilms before and after treatment with red thyme (B) or white thyme (B) laponite gel at 8 × MIC. Gels were applied at 24h. [] denotes SEM. *N* (biological repeat) = 2, *n* (technical repeat) = 2

	***P. aeruginosa***			
**Time (h)**	**Red thyme gel**		**White thyme gel**	
	**Log10 CFU**	**Log change vs T24**	**Log10 CFU**	**Log change vs T24**
**24**	8.49 [+ / − 0.57]	N/A	8.84 [+ / − 0.41]	N/A
**48**	6.16 [+ / − 1.2]	− 2.33	5.90 [+ / − 0.073]	− 2.94
**72**	5.98 [+ / − 0.56]	− 2.51	6.44 [+ / − 0.005]	− 2.40
	***S. aureus***			
**Time (h)**	**Red thyme gel**		**White thyme gel**	
	**Log10 CFU**	**Log change vs T24**	**Log10 CFU**	**Log change vs T24**
**24**	8.57 [+ / − 0.38]	N/A	8.59[+ / − 0.17]	N/A
**48**	6.21 [+ / − 0.48]	− 2.36	5.88 [+ / − 0.34]	− 2.71
**72**	6.37 [+ / − 0.62]	− 2.20	6.10 [+ / − 0.56]	− 2.49

## Discussion

Essential oils from the *Lamiaceae* family are well recognised for their antimicrobial activity against wound pathogens in vitro, yet none are licensed for clinical use. Barriers to translation include variable chemical composition, lack of standardised formulations, and concerns about toxicity (Gheorghita et al. 2022; Khwaza and Aderibigbe 2025; Roshni and Rekha 2024; Visan and Negut 2024). Despite these challenges, their multimodal mechanisms of action make them promising candidates for wound care, with a lower risk of promoting antimicrobial resistance (Nieto 2017).

We evaluated ten *Lamiaceae* essential oils for inhibitory activity and characterised their composition using TD-GCMS. Only two oils (red thyme B and white thyme B) met the criteria for classification as antimicrobial essential oils, with MICs between 100 and 1000 µg/mL (Bubonja-Šonje et al. 2020). A search of the published literature reveals highly variable MIC values for oils of the *Lamiaceae* family, indicating an assortment of antimicrobial, bactericidal or bacteriostatic activity (Ghavam et al. 2022; György et al. 2023; Kumar et al. 2016; Ramos Da Silva et al. 2021). In our study, seven oils demonstrated bactericidal activity against both *P. aeruginosa* and *S. aureus*. Consistent with previous reports, thyme oils were the most effective at low dilutions (Brożyna et al. 2024; Fournomiti et al. 2015; Sakkas and Papadopoulou 2017). Oregano and basil oils were less potent, contrary to some studies (Helal et al. 2019; Hirsch et al. 2024), while clary sage (B) and rosemary (B) showed lower efficacy overall.

Chronic wounds are invariably colonised by bacterial biofilms, meaning any essential oils that might be used as topical wound treatments must have anti-biofilm activity (Percival et al. 2015). Clary sage (B), red thyme (A and B), and white thyme (A and B) prevented *S. aureus* biofilm development, with a similar pattern observed for *P. aeruginosa* except that white thyme (A) was ineffective. Other oils reduced biofilm biomass to varying degrees, but these effects did not correlate with MIC or MBC values, highlighting the different mechanisms of action involved. Numerous essential oils are reported to have antibiofilm activity, and of these thyme has been best studied at the molecular level, with cellular targets, such as LasR and RlhR (essential for the biofilm lifestyle) identified by molecular docking (Ramos Da Silva et al. 2021; Touati et al. 2025).

TD-GCMS analysis was used to determine the chemical composition of all ten oils. Six oils (basil, clary sage (A and B), oregano, rosemary (A), and white thyme (A)) contained the emollients isopropyl myristate and/or isopropyl palmitate (Vadgama et al., 2015). These typically exhibited higher MICs (≥ 37.5 mg/mL). Notably, white thyme (A), the only thyme oil containing an emollient, had substantially higher MICs than other thyme oils, suggesting that emollients may reduce antimicrobial efficacy, as is the case when essential oils are diluted in synthetic carrier oils prior to skin application (Orchard et al. 2019).

The most abundant compounds (> 10% composition (De Sousa et al. 2023)) varied widely across oils. All thyme oils contained at least one thymol isomer, with red thyme (A) additionally containing *o*-cymene, a compound found in other antimicrobial plants such as cumin (Wanner et al. 2010). Despite belonging to the same genus (*Salvia*), clary sage (A and B) and rosemary (A and B) had distinct chemical profiles: clary sage oils were rich in linalool and linalyl acetate, whereas rosemary (B) contained high levels of 3-carene, camphor, and eucalyptol. Although these major compounds are terpenes or terpenoids, typically classed as strong antibacterial agents, they did not uniformly confer antimicrobial potency (Ergüden 2021; Guimarães et al. 2019).

It is understood that low-abundance compounds contribute to the overall antimicrobial activity of essential oils (Brandes et al. 2024; Miladinović et al. 2021). Our MIC/MBC and MBIC data indicate that the inhibitory and bactericidal activity we observed is reliant on these minor components. TD-GCMS analysis revealed considerable compositional variation in low-abundance compounds, even between oils from the same genera and species. If attributed to differing plant parts, extraction methods, and post-extraction modification, there is potential for this variability to be controlled (Fatima et al. 2025; Khalid 2025; Shahini et al. 2025; Sugier et al. 2025). However environmental factors such as temperature, pH, and water availability also affect essential oil composition (Dobhal et al. 2024; Tursun 2022; Vaičiulytė et al. 2022). These challenges mean that in practice it is difficult to produce a standardised oil with defined antimicrobial activity. Despite this, our meta-analysis effectively revealed clustering of essential oils that allied efficacy with composition, providing valuable insights that could in future could have predictive power for rapidly screening essential oils. However, these approaches do not capture the well-known synergistic, additive or antagonistic interactions among the various chemical compounds (Vaou et al. 2022).

Oils alone are unsuitable as wound treatments due to poor adherence and rapid evaporation. Gel formulations are preferable, with alginate and synthetic clays (e.g. laponite) commonly used as carriers (Abourehab et al. 2022; Do Nascimento et al. 2025; Zhou et al. 2024). For example, laponite gels have been used to deliver eugenol to wounds containing MRSA (Zhou et al. 2024). In previous work, we demonstrated that laponite gels containing hypochlorous acid or N-chlorotaurine retained antibacterial activity in both static and dynamic biofilm models (Khalid et al. 2023; Nedelea et al. 2021; Thomas et al. 2025). Building on this, we formulated laponite gels containing red thyme (B) and white thyme (B) oils based on their strong antimicrobial and antibiofilm activity, and equal MBIC values for both bacteria, which are crucial when considering topical formulations that might impair wound biofilms. Because essential oils are immiscible in hydrogels, higher concentrations were required for efficacy (Goudoulas et al. 2022).

Based on our data, we predicted that red thyme (B) gel would be most effective. Contrary to expectation, white thyme (B) gel consistently outperformed the red thyme (B) gel. Red thyme oil (B) (*T. vulgaris*) contained predominantly *o*-cymene and *o*-thymol, while white thyme oil (B) (*T. zygis*) contained high levels of *o*-thymol and thymol, and only minor *o*-cymene (3.2%). We hypothesise that the high *o*-cymene content of red thyme (B) oil, being almost water insoluble (National Institutes of Health (NIH) PubChem Database. O-Cymene | C10H14 | CID 10703), limited diffusion of the more soluble thymol isomers, which is consistent with reports of poor solubility restricting topical delivery (Folle et al. 2024; Shukr and Metwally 2014). Strategies such as PEG emulsifiers or nanoparticle systems can enhance essential oil dispersion but rarely account for compositional influences on bioavailability (Alsakhawy et al. 2022; Singh et al. 2022).

The predictive value of in vitro antimicrobial and antibiofilm assays remains limited by their reductionist design, which often fails to reflect clinical complexity. Consequently, many promising antimicrobials perform poorly in real-world contexts, as highlighted by Cochrane review (Vermeulen et al. 2007) and NICE evidence summary (NICE, 2016) for advanced wound dressings and antimicrobial dressings. We therefore tested the thyme oil gels in a realistic chronic wound biofilm model, previously shown to replicate the wound environment and the clinical performance of commercial dressings (Duckworth et al. 2020, 2018; Khalid et al. 2023; Nedelea et al. 2021). Although static assays predicted superior activity for white thyme (B) gel, both gels achieved similar log-reductions 24 h post-treatment, that were maintained at 48 h post-treatment. This contrasts with conventional wound treatments (Nedelea et al. 2021) and may reflect the sustained release provided by the immiscibility of essential oils within hydrogels (Alven et al. 2022). The differing outcomes between static and dynamic models highlight how environmental realism profoundly affects the apparent efficacy of topical antimicrobials, a key consideration for clinical translation. Encouragingly, preclinical studies in rodents indicate that thyme hydrogels can accelerate wound contraction, although this remains to be confirmed in infected wound models where bactericidal concentrations are critical (Alsakhawy et al. 2022).

## Conclusion

This study moves essential oil research beyond reductionist in vitro testing by linking detailed chemical composition with functional efficacy in a realistic wound model. By showing that the antimicrobial activity of essential oils from the same family varies widely and depends not only on major compounds but also on low-abundance components, formulation context, and delivery system, we provide insight into relevant selection criteria for efficacious essential oils. The demonstration that laponite–thyme oil gels retain sustained antibacterial and antibiofilm activity highlights their potential as practical, resistance-resilient wound treatments. Collectively, these findings offer an approach for the rational development and evaluation of natural antimicrobial formulations with clinical relevance.

## Data Availability

*data sets can be accessed on request from SH*.
